# Crystallinity-dependent transformation of layered manganese oxides: implications for the mineral diversity of manganese oxides in nature

**DOI:** 10.1093/nsr/nwag392

**Published:** 2026-07-01

**Authors:** Ke Wen, Yiping Yang, Shan Li, Jiaxin Xi, Ruiqin Yi, Hongping He, Jianxi Zhu

**Affiliations:** State Key Laboratory of Deep Earth Processes and Resources, Guangzhou Institute of Geochemistry, Chinese Academy of Sciences, Guangzhou 510640, China; Center for Advanced Planetary Science, Guangzhou Institute of Geochemistry, Chinese Academy of Sciences, Guangzhou 510640, China; Guangdong Provincial Key Laboratory of Mineral Physics and Materials, Guangzhou Institute of Geochemistry, Chinese Academy of Sciences, Guangzhou 510640, China; Guangdong Research Center for Strategic Metals and Green Utilization, Guangzhou 510640, China; State Key Laboratory of Deep Earth Processes and Resources, Guangzhou Institute of Geochemistry, Chinese Academy of Sciences, Guangzhou 510640, China; Center for Advanced Planetary Science, Guangzhou Institute of Geochemistry, Chinese Academy of Sciences, Guangzhou 510640, China; Guangdong Provincial Key Laboratory of Mineral Physics and Materials, Guangzhou Institute of Geochemistry, Chinese Academy of Sciences, Guangzhou 510640, China; Guangdong Research Center for Strategic Metals and Green Utilization, Guangzhou 510640, China; State Key Laboratory of Deep Earth Processes and Resources, Guangzhou Institute of Geochemistry, Chinese Academy of Sciences, Guangzhou 510640, China; Center for Advanced Planetary Science, Guangzhou Institute of Geochemistry, Chinese Academy of Sciences, Guangzhou 510640, China; Guangdong Provincial Key Laboratory of Mineral Physics and Materials, Guangzhou Institute of Geochemistry, Chinese Academy of Sciences, Guangzhou 510640, China; Guangdong Research Center for Strategic Metals and Green Utilization, Guangzhou 510640, China; State Key Laboratory of Deep Earth Processes and Resources, Guangzhou Institute of Geochemistry, Chinese Academy of Sciences, Guangzhou 510640, China; Center for Advanced Planetary Science, Guangzhou Institute of Geochemistry, Chinese Academy of Sciences, Guangzhou 510640, China; Guangdong Provincial Key Laboratory of Mineral Physics and Materials, Guangzhou Institute of Geochemistry, Chinese Academy of Sciences, Guangzhou 510640, China; Guangdong Research Center for Strategic Metals and Green Utilization, Guangzhou 510640, China; State Key Laboratory of Deep Earth Processes and Resources, Guangzhou Institute of Geochemistry, Chinese Academy of Sciences, Guangzhou 510640, China; Center for Advanced Planetary Science, Guangzhou Institute of Geochemistry, Chinese Academy of Sciences, Guangzhou 510640, China; State Key Laboratory of Deep Earth Processes and Resources, Guangzhou Institute of Geochemistry, Chinese Academy of Sciences, Guangzhou 510640, China; Center for Advanced Planetary Science, Guangzhou Institute of Geochemistry, Chinese Academy of Sciences, Guangzhou 510640, China; Guangdong Provincial Key Laboratory of Mineral Physics and Materials, Guangzhou Institute of Geochemistry, Chinese Academy of Sciences, Guangzhou 510640, China; Guangdong Research Center for Strategic Metals and Green Utilization, Guangzhou 510640, China; University of Chinese Academy of Sciences, Beijing 100049, China; State Key Laboratory of Deep Earth Processes and Resources, Guangzhou Institute of Geochemistry, Chinese Academy of Sciences, Guangzhou 510640, China; Center for Advanced Planetary Science, Guangzhou Institute of Geochemistry, Chinese Academy of Sciences, Guangzhou 510640, China; Guangdong Provincial Key Laboratory of Mineral Physics and Materials, Guangzhou Institute of Geochemistry, Chinese Academy of Sciences, Guangzhou 510640, China; Guangdong Research Center for Strategic Metals and Green Utilization, Guangzhou 510640, China; University of Chinese Academy of Sciences, Beijing 100049, China

**Keywords:** Mn oxides, structural transformation, crystallinity, mineral diversity, metal cycling

## Abstract

Manganese (Mn) oxides are crucial for the cycling of trace metals and nutrients. Over 30 types of Mn oxide minerals with distinct crystal structures have been identified across a variety of biogeochemical settings, yet the mechanisms underlying their formation are poorly understood. By incubating three layered Mn oxides of varying particle size and crystallinity under identical solution conditions, we observed the formation of distinct tunneled Mn oxides. The nanoscale mineral property, particularly vacancy density and surface energy, modulates the adsorption geometry of Mn(II) and subsequent electron transfer and structural rearrangement. These findings indicate that mineral structural properties associated with crystallinity are critical and previously underappreciated for the transformation of Mn oxides. Our study provides a mechanistic basis for understanding the natural diversity of Mn oxide minerals, suggesting that they likely derive from the transformation of biogenic layered Mn oxides that experienced dynamic variations in mineral crystallinity and local environmental conditions.

## Teaser

Incubation of layered birnessite of varying crystallinity and particle size with aqueous Mn(II) yields distinct tunneled Mn oxide phases, uncovering a previously overlooked control of crystallinity-related properties on mineral transformation. This discovery redefines our understanding of Mn oxide mineral diversity and its influence on elemental cycling across terrestrial and marine environments.

## INTRODUCTION

Manganese (Mn) oxides are ubiquitous in terrestrial and aquatic settings, including soils, freshwater, and marine sediments [1–3]. Over 30 distinct Mn oxide phases with varying crystal structures and chemical compositions have been identified in nature, among which birnessite-like layered Mn oxides are the most common [1]. These layered Mn oxides represent the primary and initial products of microbial-mediated oxidation of Mn(II) in a variety of environments [4]. It is because homogeneous abiotic Mn(II) oxidation by atmospheric O_2_ proceeds negligibly in ambient conditions [5], unless it is facilitated through catalytic processes on surfaces of minerals such as iron oxides, titanium oxides, and phyllosilicates [6–8]. Owing to their large surface area, high oxidizing potential of structural Mn(IV), and abundant Mn(IV) vacancies, layered Mn oxides exhibit exceptional adsorption and oxidative capacities. Accordingly, they act as metal scavengers and mineral oxidants in the environment, regulating the cycling of trace metals, nutrient availability, carbon sequestration, and the fate of contaminants [9,10].

The high reactivity of birnessite stems from its layered structure, abundant structural defects, and high oxidizing potential of structural Mn(IV) [11,12]. Upon interaction with reducing species such as Mn^2+^ and Fe^2+^, birnessite can undergo reductive transformations into Mn(III)-rich and tunneled Mn oxide phases, which thereby remarkably alters the metal sorption capacity and oxidative reactivity of Mn oxides [13–15]. These reductive transformation processes of birnessite commonly occur in environments with redox fluctuations and gradients, such as oxic-anoxic transition zones in soils and sediments, water columns, and oceanic hydrothermal vents [15–18]. For instance, the reductive transformation of layered birnessite to tunneled todorokite has recently been found to control the elemental cycling and isotopic composition of nickel in marine systems, through adsorption and incorporation processes, highlighting the crucial role of mineralogical evolution of Mn oxides in regulating transition metal dynamics [18].

The interaction of layered birnessite with reducing compounds is complex, because many environmental factors can significantly influence its reductive transformation pathway and kinetics, and resulting mineral phases. Previous studies have shown that solution chemistry, including parameters such as the ratio of Mn(II)/MnO_2_, pH, cation composition and concentration, presence of oxyanions, and ionic strength, plays a critical role in determining the transformation pathway and its resulting mineral products [19–23]. For example, monovalent cations (e.g. Li^+^, Na^+^, and K^+^) can promote the transformation of layered birnessite into a tunneled (4 × 4) phase via a triclinic birnessite intermediate [23]. In contrast, divalent cations (e.g. Mg^2+^, Ca^2+^) stabilize the layered triclinic intermediate and inhibit its subsequent transformation to tunneled structures. Surface-adsorbed Cu^2+^ can even prevent the hexagonal-to-triclinic transition [23]. Oxyanions such as silicate and phosphate also inhibit Mn(II)-induced reductive transformation by blocking reactive sites on birnessite through adsorption at layer edge sites or formation of Mn(II, III)–oxyanion ternary complexes at vacancies [21]. Under high Mn(II)/MnO_2_ ratios and near-neutral pH (6.0–8.5), the layered birnessite transforms into low-valence Mn oxide phases like hausmannite, groutite, feitknechtite, and manganite [20]. Under highly acidic conditions (pH ≤ 4), it transforms into tunneled phases such as nsutite (1 × 1 and 1 × 2 intergrowth), ramsdellite (1 × 2), and cryptomelane (2 × 2) [19]. At low Mn(II)/MnO_2_ ratios, triclinic birnessite forms in alkaline conditions as the product of Mn(II)-driven reductive transformation of layered birnessite [14], whereas a 4 × 4 tunneled phase prevails in circumneutral pH (6.0–8.0) [21–24].

While considerable attention has been given to the influence of solution chemistry on the interactions between aqueous Mn(II) and birnessite, the role of mineral crystallinity in this process remains poorly understood. Crystallinity is a fundamental characteristic that affects the reactivity and thermodynamic stability of minerals [25]. Biogenic Mn oxides, which resemble birnessite-like minerals and are likely the origin of most Mn oxides in the environment, typically occur as nanoparticles composed of thin stacks of MnO_6_ sheets with high vacancy densities [26]. Compared to the synthetic analogues, biogenic birnessite exhibit greater adsorption and redox reactivity due to their larger specific surface area and abundance of reactive edge sites and structural vacancies [27,28]. After formation, biogenic birnessite nanoparticles may undergo structural transformation and compositional evolution depending on biogeochemical conditions [29]. Interactions with environmental components can further modulate the crystallinity and particle size of birnessite, thereby influencing its environmental behavior [21]. However, whether and how mineral crystallinity influences the Mn(II)-induced transformation of layered birnessite and resulting products remain unexplored. This knowledge gap limits our understanding of the evolution and functions of Mn oxides in the environment.

In this study, we investigated the effects of crystallinity on the reductive transformation of layered Mn oxides through the incubation of synthetic birnessite with aqueous Mn^2+^. Three types of birnessite, i.e. polymeric birnessite (poly-bir), δ-MnO_2_ (delta-bir), and acid birnessite (acid-bir) were synthesized as analogues to biogenic Mn oxides with varying crystallinity and particle sizes. The morphological and structural characteristics of the layered Mn oxide precursors and their transformation products were characterized using transmission electron microscopy (TEM) and synchrotron radiation X-ray diffraction (SR-XRD). This work aims to provide new insights into the structural evolution and mineral diversity of Mn oxides, thereby enhancing our understanding of their functional roles in environmental processes.

## RESULTS

**Figure 1.**
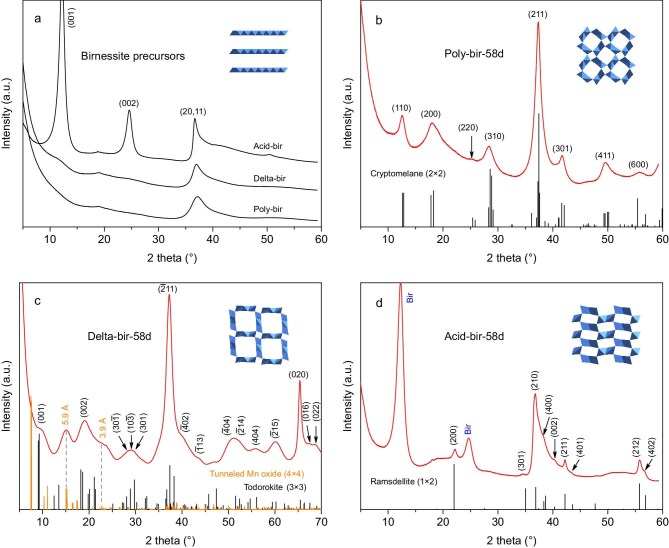
Synchrotron radiation X-ray diffraction (SR-XRD) patterns of the synthetic birnessite precursors of varying particle sizes and crystallinity (a), and their reductive transformation final products (b–d) derived from incubations with aqueous Mn at Mn(II)/MnO_2_ molar ratio of 4.26 and pH 4 for 58 days. Data were converted to Cukα radiation (λ = 1.5406 Å) data for comparison with mineral references. The arising of diffraction peaks at ∼5.9 and 3.9 Å in the pattern of delta-bir-58d (c) is ascribed to the presence of 4 × 4 tunneled structural Mn oxide, a mineral phase reported in our previous work about the Mn(II)-induced reductive transformation of layered Mn oxides [21–24].

The SR-XRD patterns of the synthesized precursors showed diffraction peaks at *d*-spacings of ∼7.2 Å, 3.6 Å, and 2.4 Å, corresponding to the (001), (002), and (20,11) reflections of layered birnessite, respectively (Fig. 1a). A small and broad feature at ∼19° (2θ) (*d* ∼ 4.6 Å) was observed in all samples, which can be assigned to the (002) reflection of feitknechtite. The presence of this phase is consistent with previous reports suggesting feitknechtite as an intermediate during the formation of birnessite-like structures [24,30]. Compared with acid-bir, the (001) and (002) reflections related to layer stacking were weakened in delta-bir and absent in poly-bir. In addition, the broad ‘hump’ around 50° (2θ), attributed to turbostratic stacking of MnO_6_ sheets, was less pronounced in delta-bir and poly-bir. The coherent scattering domain (CSD) size in the *ab* plane was determined using the Scherrer equation based on peak profile fitting of the (20,11) reflection (Fig. S1). The resulting CSD sizes were 50, 63, and 92 Å for poly-bir, delta-bir, and acid-bir, respectively (Table 1). The CSD size along *c*-axis was obtained from fitting of the (001) reflection (Fig. S1), reflecting the stacking extent of MnO_6_ sheets [31]. The *c*-axis CSD sizes for poly-bir, delta-bir, and acid-bir were 48, 57, and 119 Å, which correspond to average stacking layer numbers of 6.68, 7.95, and 16.53, respectively (Table 1).

**Table 1.** Properties of the synthetic birnessite precursors.

	FWHM*_ab_* (°)	CSD*_ab_* (Å)	FWHM*_c_* (°)	CSD*_c_* (Å)	Number of layers	BET (m^2^/g)	*D*_h_ (nm)
Poly-bir	0.82	50.53	0.87	48.08	6.68	233	142
Delta-bir	0.66	63.31	0.73	57.21	7.95	174	220
Acid-bir	0.45	92.27	0.35	119.00	16.53	45	460

FWHM*_ab_*: the full width at half maximum of the (20,11) diffraction peak, which is to represent the structure and size of the octahedral layer in the *ab* plane

CSD*_ab_*: the coherent scattering domains in the *ab* plane

FWHM*_c_*: the full width at half maximum of the (001) diffraction peak, which reflects the stacking extent of the octahedral layers perpendicular to the ab plane

CSD*_c_*: the coherent scattering domains along the axis perpendicular to the *ab* plane

number of layers: the number of MnO_6_ octahedral layers estimated according to the CSD*_c_* and an assumption of basal *d*_(001)_ distance of 7.20 Å for hexagonal birnessite

BET: the specific surface area determined using the N_2_ adsorption-desorption method

*D*_h_: the hydrodynamic particle size evaluated using the dynamic light scattering method.

In the absence of aqueous Mn(II), the layered framework of birnessite remained stable with no discernible structural changes after 58 days of incubation at pH 4 (Fig. S2). In contrast, incubation with aqueous Mn(II) at pH 4 for 58 days induced the reductive transformation of the three layered birnessite precursors, i.e. poly-bir, delta-bir, and acid-bir, into cryptomelane (2 × 2), todorokite (3 × 3), and ramsdellite (1 × 2), respectively (Fig. 1b–d). The SR-XRD pattern of poly-bir-58d matches well with the reflections corresponding to cryptomelane (Fig. 1b), confirming it as the dominant phase in the transformation products of poly-58d. The diffraction peak at 9.6 Å (2θ = 9.3°) in the SR-XRD pattern of delta-bir-58d is diagnostic of todorokite and/or buserite (Fig. 1c), corresponding to the (001) reflection. Peaks at ∼5.9 Å and ∼3.9 Å arise likely due to the presence of a 4 × 4 tunneled Mn oxide phase in the delta-bir transformation products (Fig. 1c), as observed in previous studies about Mn(II)-induced transformation of δ-MnO_2_ [21–24]. For acid-bir, the SR-XRD pattern at 58 days revealed the formation of ramsdellite, given the matches of the diffraction peaks (Fig. 1d). The sharp diffraction peaks at 7.2 Å (2θ = 12.3°) and 3.6 Å (2θ = 24.8°) correspond to the (001) and (002) basal reflections of layered birnessite, respectively.

**Figure 2.**
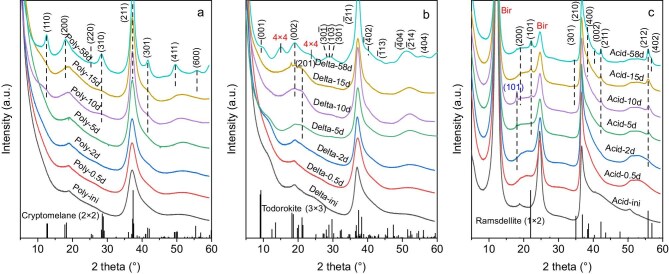
Synchrotron radiation X-ray diffraction (SR-XRD) patterns of the synthetic birnessite precursors of varying particle sizes and crystallinity, and their reductive transformation products collected at various incubation time intervals during incubation with aqueous Mn at Mn(II)/MnO_2_ molar ratio of 4.26 and pH 4 for 58 days. (a) Poly-bir, (b) delta-bir, and (c) acid-bir.

Time-resolved SR-XRD patterns revealed the progressive structural evolution of the layered birnessite precursors during incubation with aqueous Mn(II) at pH 4. For poly-bir, no significant changes were observed until 10 days, when new diffraction peaks appeared at 2θ = 12.7°, 28.4°, and 41.6°, corresponding to the (110), (310), and (301) reflections of cryptomelane (Fig. 2a). These cryptomelane reflections intensified with continued incubation, while additional reflections including (200), (220), (211), (411), and (600) emerged by 58 days (Figs 1b and 2a). No other Mn oxide phases were detected in the poly-bir transformation products at any selected sampling time. For delta-bir, formation of todorokite was first indicated at 5 days by the rise of a broad (201) reflection at 2θ = 21.1° (Fig. 2b). At 10 days, a new diffraction peak was observed at 2θ = 19.0°, corresponding to the todorokite (002) reflection. A suite of todorokite reflections appeared at 58 days, including (001), (002), (30${\bar{1}}$), (10${\mathrm{\bar{3}}}$), (301), (${\mathrm{\bar{2}}}$11), (${\mathrm{\bar{4}}}$02), (${\mathrm{\bar{1}}}$13), (${\mathrm{\bar{4}}}$04), (${\mathrm{\bar{2}}}$14), (404), (${\mathrm{\bar{2}}}$15), (020), (016), and (022) (Figs 1c and 2b). However, the (201) reflection of todorokite at 2θ = 21.1° disappeared at 58 days, while two new peaks at ∼5.9 Å and ∼3.9 Å indicated the presence of a 4 × 4 tunneled Mn oxide phase (Figs 1c and 2b). For acid-bir, the transformation products throughout the 58-day incubation were dominated by well-crystallized layered birnessite, as indicated by the sharp (001) and (002) basal reflections (Fig. 2c). The formation of ramsdellite was first identified at 5 days, based on the (101), (211), and (212) reflections, followed by the (200) reflection at 10 days and additional reflections such as (301), (400), (002), and (402) at 15–58 days. A transient diffraction peak at 2θ = 18.0° appeared at 2 days, intensified until 10 days, and then disappeared by 15–58 days. This peak corresponds to the (101) diffraction of hausmannite (Mn_3_O_4_), suggesting that hausmannite likely occurred as an intermediate phase during the Mn(II)-induced transformation of layered birnessite into the tunneled ramsdellite.

**Figure 3.**
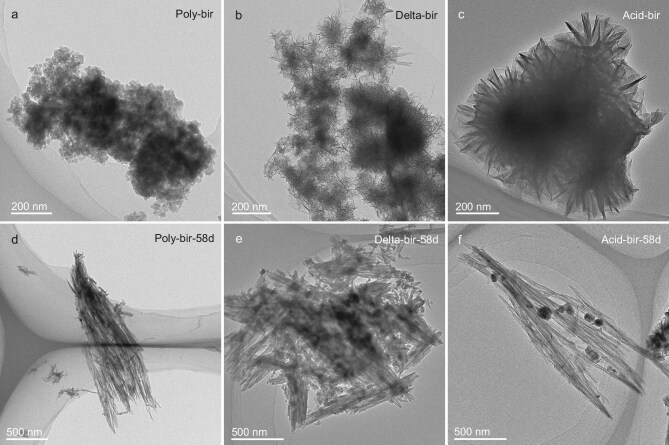
TEM images of the synthetic birnessite precursors of varying partile sizes and crystallinity (a–c), and their reductive transformation products (d–f) derived from the incubation of birnessite with aqueous Mn(II) at Mn(II)/MnO_2_ molar ratio of 4.26 at pH 4 for 58 days.

TEM observations corroborated the SR-XRD data, confirming the successful synthesis of three-layered birnessite precursors by showing the similar nanoflake morphology of three types of precursors (Fig. 3a–c). The ‘nanoflower’ morphology, resulting from the folding of thin MnO_2_ octahedral sheets at edges [27], was observed in delta-bir (Fig. 3b) and even more pronounced in acid-bir (Fig. 3c), but was absent in poly-bir (Fig. 3a). Following incubation with aqueous Mn(II) at pH 4 for 58 days, the transformation products of three birnessite precursors exhibited a needle-like morphology (Fig. 3d–f). The original nanoflake (or nanoflower) morphology disappeared in poly-bir-58d (Fig. 3d) and delta-bir-58d (Fig. 3e), whereas it was partially retained in acid-bir-58d (Fig. 3f). HRTEM images and corresponding FFT patterns exhibited distinct lattice fringes with *d*-spacings of 4.9, 2.5, and 1.8 Å in poly-bir-58d, which were assigned to cryptomelane (Fig. S4a–c); 7.0, 3.2, 2.4, and 1.4 Å in delta-bir-58d, corresponding to todorokite (Fig. S4d–f); and 4.6, 2.5, and 1.4 Å in acid-bir-58d, corresponding to ramsdellite (Fig. S4g–i). These identifications of mineral phases were further demonstrated by the selected area electron diffraction (SAED) patterns and corresponding profiles with comparison with mineral references (Fig. S5).

**Figure 4.**
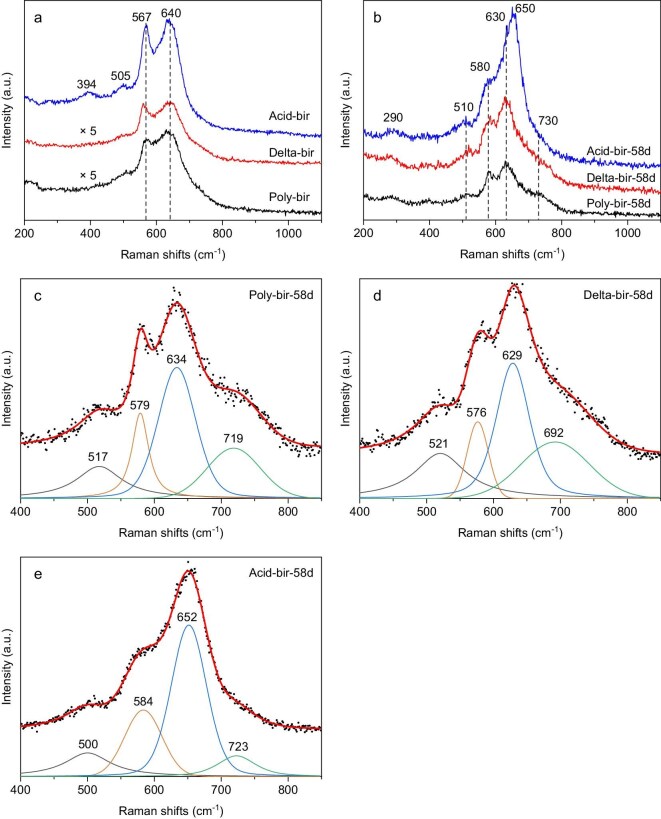
Raman spectra of the synthetic birnessite precursors of varying particle sizes (a), and their reductive transformation products (b). Raman spectra of the transformation products at the range of 400–850 cm^−1^ were fitted to quantitively determine the differences among three samples (c–e). The 650 cm^−1^ band in acid-58d (b and e) likely arises due to the presence of hausmannite as transformation intermediate at the 58 days incubation.

Raman spectroscopy provided complementary structural characterization of the Mn oxides before and after incubation. All three precursors exhibited two characteristic high-frequency bands at 567 cm^−1^ and 640 cm^−1^, while two additional low-frequency bands at 394 cm^−1^ and 505 cm^−1^ were observed only for acid-bir (Fig. 4a). Compared with acid-bir, these two high-wavenumber bands are broadened and attenuated in poly-bir and delta-bir, while the low-wavenumber bands are absent (Fig. 4a). After incubation with aqueous Mn(II) at pH 4 for 58 days, the Raman spectra of the transformation products showed two main high-frequency bands at ∼580 cm^−1^ and ∼630 cm^−1^, along with two weak bands at ∼510 cm^−1^ and ∼730 cm^−1^ (Fig. 4b). Peak fitting of the 400–850 cm^−1^ region revealed four bands for each sample: 517, 579, 634, and 719 cm^−1^ for poly-bir-58d (Fig. 4c); 521, 576, 629, and 692 cm^−1^ for delta-bir-58d (Fig. 4d); and 500, 584, 652, and 723 cm^−1^ for acid-bir-58d (Fig. 4e).

## DISCUSSION

### Characteristics of birnessite precursors and transformation products

**Figure 5.**
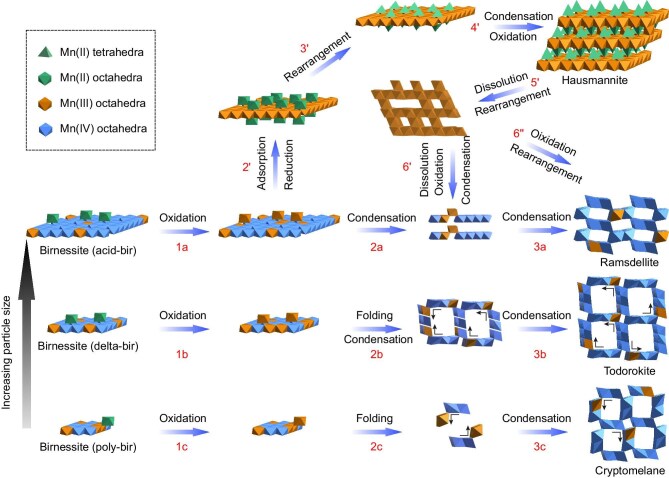
Schematic illustrating the potential transformation pathways from three layered birnessite precursors of varying particle sizes, into distinct tunneled Mn oxides. Under strong acidic conditions, the adsorbed Mn(II) either on vacancies or edge sites of layered Mn oxides, undergoes oxidation (path 1) by structural Mn(IV), which produces two Mn(III). The presence of structural abundant Mn(III) on edge sites and vacancies reduces the stability of the layered Mn oxides, causing transformations into distinct tunneled Mn oxide phases through folding and/or structural condensation mechanisms (path 2 & 3). Abundant aqueous Mn(II) adsorbed onto vacancies can lead to substantial reduction of structural Mn(IV) to be Mn(III), resulting a Mn(III, II)-baring phase (path 2’). The excess low-valance Mn(II) and Mn(III) destabilizes the layered structure of birnessite, which leads to the formation of hausmannite via structural rearrangement and condensation (path 3’ & 4’). Hausmannite occurs as an intermediate phase due to the strong acidic conditions, and its subsequent dissolution and oxidation causes formation of tunneled Mn oxides through structural rearrangement and condensation (path 5’, 6’, and 6’’).

The three precursors, i.e. poly-bir, delta-bir, and acid-bir, exhibit a similar disk-like morphology (Fig. 3a–c), despite being synthesized via different methods. This morphological similarity is consistent with the presence of (20,11) reflection in the SR-XRD patterns (Fig. 1a), indicating a layered structure of three precursors. The absence of splitting of the (20,11) reflection (Fig. 1a), which distinguishes orthogonal from hexagonal layer symmetry [32,33], further confirms that all three precursors share the same hexagonal layer symmetry. However, differences are evident in the layer stacking extent and crystallinity. Compared with acid-bir, the broadened and attenuated (001) and (002) reflections in delta-bir and poly-bir (Fig. 1a) likely suggest reduced structural coherence along the *c*-axis and lower crystallinity. This interpretation is supported by the increased full width at half maximum of the (001) and (20,11) reflections (Fig. S1) and the corresponding decrease in CSD sizes (Table 1). Given that the CSD size in the *ab* plane represents the lateral dimension of MnO_6_ octahedral sheets, whereas that along the *c*-axis reflecting the layer stacking extent [31], the systematic increase in CSD sizes in both directions (Table 1) indicate an increase in particle size and crystallinity in the order of poly-bir < delta-bir < acid-bir. This order is further corroborated by the decrease in specific surface area from 233 to 174 and to 45 m^2^/g, and the increase in hydrodynamic particle size from 142 to 220 and to 460 nm (Table 1 and Fig. S6). The more intense diffraction rings in SAED (Fig. S3), and sharper and more intense Raman bands for acid-bir provide additional evidence for its higher degree of crystallinity compared to poly-bir and delta-bir (Fig. 4a). In Raman spectra, the band at 640 cm^−1^ corresponds to the symmetric stretching vibration of MnO_6_ octahedra along the *c*-axis, while the band at 567 cm^−1^ is primarily associated with the in-plane shear motion of O atoms within the MnO_6_ sheets [34,35]. These bands are sensitive to ionic substitution, structural alteration, and the type and concentration of interlayer cations [34–37]. Notably, the band around 640 cm^−1^ has been reported to shift linearly to lower wavenumbers with increasing ratio of Mn(III)/(Mn(III)+Mn(IV)) [34]. Given the similar layered structures (Fig. 1a), comparable interlayer cation compositions, and nearly identical band positions at ∼640 cm^−1^ (Fig. 5a), the three precursors likely contain a similar Mn(III) fraction of ∼35%, based on the reported linear relationship [34].

After incubation with Mn(II) under identical conditions for 58 days, the three precursors evolve to distinct tunneled Mn oxide phases depending on their initial particle size and crystallinity: poly-bir transforms into cryptomelane (2 × 2), delta-bir into todorokite (3 × 3), and acid-bir into ramsdellite (1 × 2) (Figs 1b–d and 2). The formation of cryptomelane from poly-bir is supported by (i) the close match between observed and reference diffraction peaks (Fig. 1b) and (ii) the characteristic lattice fringes (Fig. S4a–c) and SAED patterns (Fig. S5a and d). Todorokite is identified as the primary transformation product of delta-bir based on the SR-XRD pattern (Fig. 1c), although the diffraction feature at ∼9.6 Å could also suggest the possible presence of buserite. However, the formation of buserite can be reasonably excluded based on several considerations. First, no divalent cations (e.g. hydrated Mg^2+^), which are typically associated with buserite interlayers [38], are present in our system. Second, the transformation products were thoroughly dehydrated prior to the SR-XRD measurements, minimizing the likelihood of humidity-induced transformation from birnessite to buserite in the absence of divalent cations [39]. Third, TEM observations reveal a needle-like morphology (Fig. 3e), characteristic of todorokite rather than the nanoflake morphology of buserite. Fourth, the observed lattice fringes (Fig. S4d–f) and SAED patterns (Fig. S5b and e) are consistent with todorokite. Finally, the diffraction peak at 2.4 Å (2θ = 37.3°), corresponding to the (${\mathrm{\bar{2}}}$11) reflection of todorokite, further distinguishes todorokite from layered Mn oxides based on its characteristic peak shape [40]. In addition, minor amounts of 4 × 4 tunneled Mn oxide likely occur in delta-bir-58d, as suggested by diffraction peaks at ∼5.9 Å and ∼3.9 Å (Fig. 1c). It is consistent with previous studies [22–24,41], although this phase was not identified by HRTEM likely due to the low abundance. The formation of ramsdellite in acid-bir-58d is confirmed by SR-XRD (Fig. 1d) as well as the identification of characteristic lattice fringes (Fig. S4g–i) and SAED patterns (Fig. S5c and f). However, a fraction of well-crystallized acid-bir persists after 58 days, as evidenced by the residual (001) and (002) basal reflections (Fig. 1d) and the nanoflake morphology observed by TEM (Fig. 3f).

Raman spectra provide additional evidence for the formation of the three distinct tunneled Mn oxide phases. The characteristic bands at 567 cm^−1^ and 640 cm^−1^ for birnessite shift to ∼580 cm^−1^ and ∼630 cm^−1^, respectively, in the transformation products (Fig. 4b), which can be ascribed to the incorporation of Mn(III) during the Mn(II)-induced reductive transformations [34,36]. Since Mn(III) has a larger ionic radius than Mn(IV), its incorporation leads to longer average Mn-O lengths and induces a Jahn-Teller distortion of MnO_6_ octahedra, thus elongating the axial bonds relative to the equatorial ones. Consequently, the high-frequency band primarily arising from the axial Mn-O stretching shifts to lower wavenumbers with increasing Mn(III) content, whereas the lower-frequency band shifts to higher wavenumbers [34,36].

Structural factors, particularly the degree of octahedral polymerization, also influence Raman band positions, especially at high wavenumbers. A general decrease in band wavenumber has been observed with increasing octahedral polymerization (i.e. the number of edges shared per MnO_6_ unit) [35,37]. The poly-bir-58d likely contains a similar Mn(III) content as delta-bir-58d, as indicated by their comparable Raman band positions (∼580 and ∼630 cm^−1^; Fig. 4b). However, quantitative analysis shows that the band positions in poly-bir-58d (Fig. 4c) are slightly higher than those in delta-bir-58d (Fig. 4d). In particular, the band above 700 cm^−1^ that attributes to the stretching vibrations of the shortest and strongest Mn(IV)-O bonds in rigid octahedral tunnel frameworks, is more pronounced in poly-bir-58d than in delta-bir-58d. The red shifts of high-wavenumber bands from poly-bir-58d to delta-bir-58d suggests an increase in octahedral polymerization. Consistently, the fraction of edge-sharing octahedra is lower in cryptomelane (0.5, 2 × 2) than in todorokite (0.636, 3 × 3) [24,37], supporting the phase assignments based on SR-XRD (Figs 1 and 2). In contrast, the blue shift of high-wavenumber bands in acid-58d (Fig. 4e) relative to poly-bir-58d (Fig. 4c) and delta-bir-58d (Fig. 4d) can be attributed to the formation of ramsdellite (1 × 2), which has a lower fraction (0.385) of edge-sharing octahedra [24]. The weak intensity of the band above 700 cm^−1^ in acid-bir-58d (Fig. 4e), reflects the likely presence of hausmannite. The band at ∼650 cm^−1^ may also be a characteristic of hausmannite that can be attributed to Mn-O breathing vibrations of tetrahedral Mn(II) in spinel structures [36,37]. In addition, partial substitution of Mn(IV) by Mn(III) may contribute to the red shift and/or attenuation of high-wavenumber bands in delta-bir-58d (Fig. 4d) and acid-bir-58d (Fig. 4e) due to softening of Mn-O stretches [34–37]. The absence of other hausmannite bands suggests that it occurs only as a minor phase, consistent with its nondetection by SR-XRD. This discrepancy likely arises from the higher sensitivity of Raman spectroscopy to local bonding environments.

### Transformation mechanism

The stability of Mn oxides in the Mn-H_2_O-O_2_ system is thermodynamically governed by pH and E_h_ conditions [3,12]. This stability can be altered through reductive dissolution and transformation triggered by the introduction of Mn(II) or changes in pH [20]. Previous studies indicate that the mineral phase derived from the reductive transformation of layered birnessite depends primarily on solution chemistry, including pH, Mn(II)/MnO_2_ molar ratio, and the composition of background electrolytes [14,20–24]. However, in this study, we observed the formation of distinct tunneled Mn oxides (Figs 1, 2, S4, and S5) after incubating layered birnessite precursors of varying particle size and crystallinity with aqueous Mn(II), even under the identical solution condition (pH 4.0, 100 mM NaCl, and Mn(II)/MnO_2_ molar ratio of 4.26). The divergent phases are attributable to the different particle size and crystallinity of the precursors. No transformation occurred in the absence of aqueous Mn(II) at pH 4 over 58 days (Fig. S2), consistent with the previous study [24]. Moreover, the oxidation of Mn(II) by atmospheric O_2_ in birnessite suspensions is negligible under such acidic conditions (pH ≤ 6) [20]. Therefore, the transformation of layered birnessite is primarily driven by redox reactions between adsorbed Mn(II) and structural Mn(IV) in the birnessite lattice (path 1 in Fig. 5), rather than by proton-promoted dissolution.

Thermodynamically, the stability and transformation of nano-minerals are influenced by the contribution of surface energy to the total free energy [42]. In large and well-crystallized birnessite (e.g. acid-bir), basal planes dominate the total large surface areas, whereas edge sites contribute to a significant portion of the surface in small and poorly crystalline particles (e.g. poly-bir). The relative abundance and contribution of reactive edge sites substantially increase for acid-bir to delta-bir to poly-bir, because of the decrease in particle size and crystallinity (Table 1 and Fig. 3a–c). Owing to the high surface–to–volume ratio of birnessite nanoparticles, surface energy contributes a large proportion of the total free energy [42]. Consequently, a decrease in crystallinity and particle size of birnessite elevates the total free energy of the system, thus reducing its thermodynamic stability and increasing its transformations. Furthermore, the electronic band structure of birnessite can be different with variations in particle size [43]. The UV-vis spectrum of poly-bir exhibited a blue shift compared to delta-bir and acid-bir (Fig. S7), indicating a wider band gap and higher intrinsic chemical reactivity. These combined effects of reduced crystallinity and particle size enhance both the thermodynamic driving force and kinetic favorability of Mn(II)-birnessite redox reactions. Thus, poly-bir underwent faster and more extensive transformation due to its higher reactivity and lower stability, whereas highly crystalline acid-bir was retained partially even at 58 days (Fig. 3f). This suggests that the structural characteristics associated with mineral crystallinity modulate both the pathway and kinetics of reductive transformation of birnessite.

As particle size increases through processes like oriented attachment of nanoparticles, abundant defects, particularly vacancies, are introduced into the structure [44]. For example, acid birnessite contains more Mn(IV) vacancies than δ-MnO_2_ [27]. Under the given solution condition, these vacancies act as the primary adsorption sites for Mn(II) in acid-bir, while edge sites dominate Mn(II) adsorption in poly-bir. This fundamental difference in the local structural environment of adsorbed Mn(II) governs the subsequent transformation pathway and the final Mn oxide phase (Fig. 5). Specifically, Mn(II) adsorbed on vacancies favors an interlayer condensation mechanism (path 2a & 3a in Fig. 5) following its oxidation by structural Mn(IV) (path 1a in Fig. 5), causing the formation of 1 × 2 (and/or 1 × 1, 1 × 3) tunneled structures. Under high Mn(II)/MnO_2_ ratio, a significant portion of structural Mn(IV) in acid-bir can be reduced by adsorbed Mn(II), causing the formation of a Mn(III, II)-bearing phase (path 2’ in Fig. 5). The excess of low-valence Mn(II) and Mn(III) destabilizes the layered structure of acid-bir, potentially leading to the formation of hausmannite through structural rearrangement and condensation (path 3’ & 4’ in Fig. 5), which involves a coordination change of structural Mn(II) from octahedral to tetrahedral (path 3’ in Fig. 5). However, under strong acidic conditions, hausmannite exists only as a transient intermediate, because it tends to dissolve, releasing Mn(II) and Mn(III). The dissolution can subsequently supply Mn(III) interlayer condensation processes (path 6’ in Fig. 5) or undergo structural rearrangement with concomitant oxidation (path 6’’ in Fig. 5) to form the 1 × 2 tunneled structure. In contrast, Mn(II) adsorbed on edge sites contributes more significantly to the formation of 2 × 2 tunneled structures, primarily through a sheet folding (path 2c in Fig. 5) mechanism followed by structural condensation (path 3c in Fig. 5). Delta-bir, which exhibits intermediate crystallinity and particle size, shows comparable contributions from both edge-adsorbed and vacancy-adsorbed Mn(II) during transformation. Consequently, the final tunneled phases arise from a combination of both structural folding and interlayer condensation mechanisms (path 2b in Fig. 5). Overall, under identical solution chemical conditions, the transformation pathway and the identity of the final products are ultimately determined by the nanoscale characteristics of the layered birnessite precursor, specifically, the abundance of vacancies.

By using ^54^Mn radiotracers, previous studies have shown that interfacial electron transfer between adsorbed Mn(II) and structural Mn(IV) drives birnessite transformation through transient Mn(III) species [45,46]. For example, feitknechtite formed during Mn(II)-birnessite interactions at pH 7, whereas only minor interlayer and sheet structural changes occurred at pH 5 even after three months [45,46]. In contrast, our results show distinct tunneled Mn oxide phases form at pH 4. This disparity is likely attributed to the higher Mn(II)/MnO_2_ ratio (4.26) employed in our work compared to previous studies (1.43) [46], which promotes the generation of abundant Mn(III) at vacancies and within the MnO_6_ sheets (Fig. 5), thereby facilitating tunneled Mn oxide formation. Consistently, Elzinga [46] noted the potential formation of minor tunneled phases like pyrolusite and nsutite at pH 5. The continuous dissolution-reprecipitation during Mn(II)-birnessite interaction produces substantial Mn(III) [46], whose stability—governed largely by pH—determines the transformation pathway of birnessite [24]. Under acidic conditions, structural Mn(III) formed at vacancies may undergo disproportionation into Mn(II) and Mn(IV), leaving behind Mn(IV) at the vacancy sites [24,46], which favors the formation of tunneled phases through olation/oxolation and structural arrangement [24].

It is well-established that the transformation of Mn oxides from layer to tunnel structures is mediated by the formation of structural Mn(III), particularly triple-corner-sharing (TCS) Mn(III) [47–49]. The nucleation of tunneled phase is proposed to initiate through the accumulation of TCS Mn(III) on layer surfaces. Our mechanistic study suggests that aqueous Mn(II) adsorbs directly onto vacancy and edge sites of birnessite, followed by oxidation via structural Mn(IV) (path 1 in Fig. 5), which produces paired Mn(III) species: one in a TCS configuration above the vacancy and the other incorporated within the MnO_6_ sheet [14,24]. Through Cs-corrected STEM, Yuan *et al.* directly observed the migration of Mn(III) that facilitated the formation of tunnels [50]. The production of TCS Mn(III) can be enhanced under acidic conditions due to two factors: (1) partial dissolution of birnessite releases Mn(II), which can re-adsorb and be oxidized, thereby replenishing TCS Mn(III); (2) promoted migration of Mn(III) from the MnO_6_ sheets to TCS surface sites, which relieves lattice strain induced by Jahn-Teller distortion [47]. The absence of transformation in control experiments without added aqueous Mn(II) (Fig. S2) confirms that the formation of TCS Mn(III), essential for initiating the transformation, stems from redox reactions between adsorbed Mn(II) and structural Mn(IV).

### Implications for Mn oxide diversity and metal cycling

**Figure 6.**
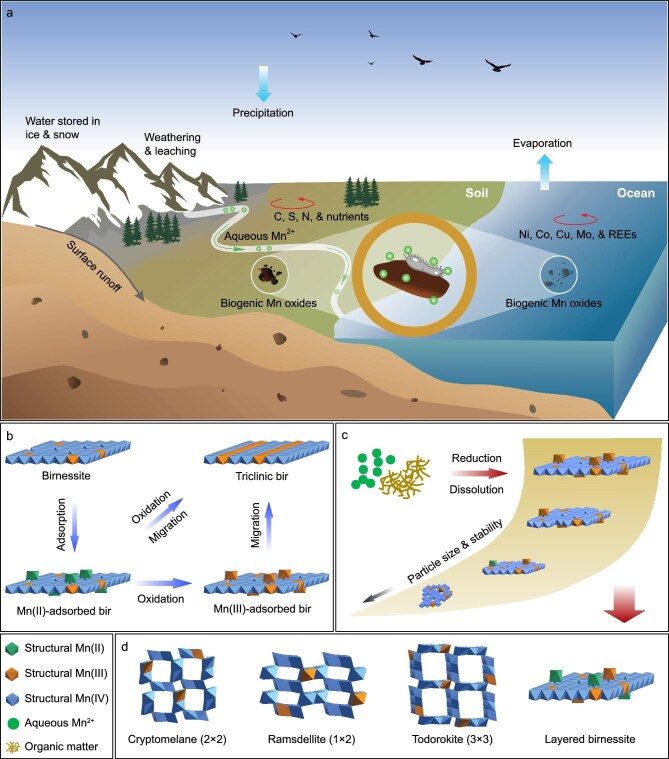
Schematic illustration of Mn redox cycling across various biogeochemical settings such as soils and marine sediments, highlighting the processes resulting in the diverse Mn oxide mineral assemblages in nature. (a) Microbial oxidation of aqueous Mn(II) initially produces birnessite-like phases as primary Mn oxides, which profoundly control the elemental cycling of nutrients, critical metals, and REEs in soils and sediments; (b) subsequent interactions with aqueous Mn(II) induces structural modifications in layered birnessite through adsorption and oxidation; (c) exposure to reducing agents in the environment, including aqueous Mn(II) and organic species, promotes reductive dissolution, decreasing particle size and crystallinity; (d) the alterations in particle size and crystallinity during the formation and dissolution of Mn oxides ultimately direct the formation of a variety of Mn oxide phases with distinct structures, even under identical chemical conditions.

Mn oxides are widely distributed in nearly all biogeochemical settings, where they regulate the cycling of nutrients, critical metals, and rare earth elements (REEs), as well as the fate of contaminants (Fig. 6a). While more than 30 distinct Mn oxide minerals have been identified in nature, layered birnessite-like minerals are the predominant phases [1]. Birnessite-like minerals have been found as key components in marine sediment nodules [2,51,52], rock varnish [53–55], soil aggregates [56,57], and they can undergo structural evolutions along with the environmental changes [15,18]. These layered Mn oxides form initially and primarily via microbially mediated oxidation of aqueous Mn(II) (Fig. 6a), because abiotic Mn(II) oxidation proceeds extremely slowly under ambient conditions [5,26]. Once released into the environment through rock-water interactions during weathering (Fig. 6a), Mn(II) can precipitate as Mn(III, IV) oxides, be transported in water bodies, and participate in many biogeochemical cycles depending on the local conditions [10]. Conversely, environmental changes such as acidification or water saturation can induce reductive dissolution of Mn oxides, releasing Mn(II) into solutions. These modifications happen mainly through adsorption, oxidation, and structural rearrangement processes (e.g. Mn(III) migration) (Fig. 6b), and thus alter the functional roles of Mn oxides in various systems.

Previous studies primarily attributed the formation of a specific Mn oxide phase to the effects of solution chemistry, including pH, Mn(II)/MnO_2_ ratio, and the composition of coexisting cations and oxyanions. In this study, distinct Mn oxides formed during the Mn(II)-induced transformation of layered birnessite, even under identical solution conditions. Results indicate that the nanoscale properties related to the mineral particle size and crystallinity, such as the density of vacancies, band structure, surface energy, governs the transformation pathway and products of layered Mn oxides. The particle size and crystallinity of layered Mn oxides can vary significantly, either during the biogenic formation of Mn oxides [26], or through subsequent interactions with aqueous Mn(II) and other reducing compounds (e.g. soil organics) in heterogeneous environments (Fig. 6c). While the primary and initial product of Mn(II) oxidation is the nanoparticulate, poorly crystalline hexagonal birnessite, its further conversion into more ordered triclinic birnessite and todorokite could be affected by microorganism species [58]. The crystallinity, crystal size, and Mn oxidation state of birnessite can be altered during redox fluctuations [59]. Once formed, acidic conditions promote the dissolution of Mn(III, IV) from edge sites, thereby reducing the particle size of birnessite [60]. Organic compounds such as humic and fulvic acids can further decrease birnessite particle size through reductive dissolution [61,62]. Ionic substitution of Mn by elements like Fe, Co, Ni, Al, and V during dissolution-reprecipitation of birnessite also modifies its crystallinity [63–65]. Taken together, we propose that the dynamic evolution of crystallinity in biogenic birnessite-like minerals, coupled with environmental heterogeneity, collectively gives rise to the diverse assemblages of Mn oxide minerals observed in nature (Fig. 6d).

The phase transformation of layered Mn oxides is accompanied by profound structural and compositional changes, which significantly affect the behavior and isotopic fractionation of various metals. Redox-sensitive elements such as Ce, Mo, Cr, Tl, and Ni serve as valuable proxies for reconstructing paleo-redox conditions and paleo-productivity, as their redox cycles are closely tied to the formation and transformation of Mn oxides [18,66–70]. The speciation, concentration, and bioavailability of critical metals (e.g. Co, Ni, Cu, Li, Mo) and REEs, which often undergo active cycling between sediment and seawater, are strongly influenced by layered Mn oxide transformations through adsorption, release, oxidation, incorporation, and precipitation processes [2,13,71–73]. For instance, during sedimentary diagenesis, the conversion of layered Mn oxides into tunnel-structured phases releases Ni into porewaters [71], whereas Co tends to be incorporated into newly formed todorokite via isomorphic substitution [13]. Conversely, the presence of these transition metals can also inhibit the transformation of layered Mn oxides into tunnel phases, leading to poorer crystallinity in the resulting tunneled Mn oxides. Overall, the crystallinity of layered Mn oxides varies dynamically throughout diverse biogeochemical processes, giving rise to a wide variety of Mn oxide minerals with distinct structures and chemical compositions. This mineralogical diversity is expected to profoundly influence the cycling of numerous metals across different biogeochemical settings, from soils to marine sediments.

## CONCLUSION

In this study, incubation of three layered birnessite precursors of varying particle size and crystallinity with aqueous Mn(II) under identical solution conditions (pH 4.0, 100 mM NaCl, and Mn(II)/MnO_2_ ratio of 4.26) induced the formation of distinct tunneled Mn oxide phases: cryptomelane (2 × 2) from poly-bir, todorokite (3 × 3) from delta-bir, and ramsdellite (1 × 2) from acid-bir. Hausmannite occurred as a transient phase during the transformation of acid-bir under a high Mn(II)/MnO_2_ ratio. These findings advance the current understanding of redox-driven transformation of layered Mn oxides, which critically control metal and nutrient cycling across diverse biogeochemical settings. While this transformation has long been believed to be determined by solution chemistry such as pH and Mn(II)/MnO_2_ ratios, our study reveals the previously overlooked role of fundamental mineral characteristics at the nanoscale, such as crystal defects, crystallinity, and surface energy associated with particle size variations, in directing the transformation pathway and products.

The present study mainly focuses on the influences of structural properties associated with crystallinity in directing the reductive transformation of Mn oxides, yet the findings are primarily based on the results under the well-controlled condition (pH 4.0, 100 mM NaCl, and Mn(II)/MnO_2_ ratio of 4.26). Future studies are warranted to examine the crystallinity-dependent transformation behavior of minerals under various pH, reductant/Mn oxide ratios, cations and oxyanions, and ionic strength. Even though, via integrating our results with prior related studies, we propose that the over 30 types of Mn oxide minerals in nature are likely derived from the redox-driven transformation of biogenic layered Mn oxides, mediated by dynamic evolutions in their crystallinity, particle size, and interactions with heterogeneous biogeochemical environments.

## METHODS

### Synthesis and transformation of birnessite

Three types of birnessite samples, i.e. acid birnessite (acid-bir), δ-MnO_2_ (delta-bir), and polymeric birnessite (poly-bir), were synthesized using established protocols in previous studies. The acid-bir was synthesized through the reduction of KMnO_4_ by HCl [74]. The delta-bir was prepared according to the method of Murray [22], by reducing KMnO_4_ with Mn(NO_3_)_2_. The poly-bir was synthesized via the reduction of KMnO_4_ by Na_2_S_2_O_3_ [75]. Transformation experiments were conducted in 300 mL suspensions, which were prepared in polyethylene bottles containing 100 mM NaCl, ∼2 g/L birnessite, and 66 mM Mn(II). The bottles were wrapped with aluminum foil to prevent light exposure. The pH was maintained at 4 throughout the incubation while being vigorously stirred. Control experiments were conducted following the same procedure but in the absence of aqueous Mn(II). All experiments were conducted in ambient air (oxic) conditions.

### Synchrotron radiation X-Ray diffraction (SR-XRD)

All ground solid samples from the incubation experiments were loaded into borosilicate glass capillary tubes of 0.5-mm outside diameter for synchrotron XRD data collection. The SR-XRD data were collected using synchrotron radiation X-rays of 0.6887 Å at the BL14B1, Shanghai Synchrotron Radiation Facility, with an exposure time of 100 s. The diffraction pattern of the empty borosilicate glass tubing was collected as the background signal, and it was subtracted from those patterns of the solid samples. The diffraction patterns of samples were converted to 1.5406 Å of Cukα radiation for comparison with the XRD data of references.

### Transmission electron microscopy (TEM)

Solid samples of initial synthetic birnessite and derived products of incubation experiments were subject to TEM imaging and SAED analyses. The characterizations were conducted at GIG-CAS using a FEI Talos F200S field-emission transmission electron microscope (ThermoFisher Scientific, USA) operated at an accelerating voltage of 200 kV. Ground samples were dispersed ultrasonically in anhydrous ethanol for 10 min. The dispersed suspension was dropped onto a carbon-coated copper microgrid and then the ethanol was allowed to evaporate prior to further observations in the TEM system. The SAED data analysis was conducted using the FEI Velox software.

### Raman spectroscopy

Raman spectra were collected at GIGCAS using a Renishaw inVia micro-Raman system (Gloucestershire, UK). Ground powders were pressed onto glass discs to obtain an approximately flat surface for Raman analysis. Areas interested were analyzed using an Olympus 50 × objective, an integration time of 50 s, and at a spectral resolution of 2 cm^−1^ using a 785 nm solid state laser coupled with a grating of 1200 gr/mm. Four acquisitions per spot were set to improve the signal-to-noise ratio. A laser power of 50 µW was applied, which is sufficiently low to avoid laser-induced damage or phase transformation of Mn oxides [34,36].

## Supplementary Material

nwag392_Supplemental_File

## Data Availability

The experimental data are available via Mendeley Data at https://doi.org/10.17632/hwhcdr945d.2.
