# Supplementary material for: Crystallinity-dependent transformation of layered manganese oxides: implications for the mineral diversity of manganese oxides in nature
Source: Natl Sci Rev. 2026 Jul 1;13(14):nwag392. doi: 10.1093/nsr/nwag392 (PMC13386507; doi:10.1093/nsr/nwag392)
Supplement: nwag392_Supplemental_File [file nwag392_supplemental_file.docx]

***Supplementary Material***

**Crystallinity-dependent transformation of layered manganese oxides: implications for the mineral diversity of manganese oxides in nature**

Ke Wen^1,2,3,4^, Yiping Yang^1,2,3,4^, Shan Li^1,2,3,4^, Jiaxin Xi^1,2,3,4^, Ruiqin Yi^1,2^, Hongping He^1,2,3,4,5^, and Jianxi Zhu^1,2,3,4,5^*

^1^State Key Laboratory of Deep Earth Processes and Resources, Guangzhou Institute of Geochemistry, Chinese Academy of Sciences, Guangzhou 510640, China.

^2^Center for Advanced Planetary Science, Guangzhou Institute of Geochemistry, Chinese Academy of Sciences, Guangzhou 510640, China.

^3^Guangdong Provincial Key Laboratory of Mineral Physics and Materials, Guangzhou Institute of Geochemistry, Chinese Academy of Sciences, Guangzhou 510640, China.

^4^Guangdong research center for strategic metals and green utilization, Guangzhou 510640, China.

^5^University of Chinese Academy of Sciences, Beijing 100049, China.

*Corresponding author. E-mail address: [zhujx@gig.ac.cn](mailto:zhujx@gig.ac.cn) (J. Zhu).

**Text S1 Synthesis of birnessite precursors**

Chemicals of A.C.S. reagent grade were purchased from Sigma-Aldrich and used as received. Solutions were prepared using deionized (DI) water with a resistivity of 18.2 MΩ·cm. Three types of birnessite samples, i.e., acid birnessite (acid-bir), δ-MnO_2_ (delta-bir), and polymeric birnessite (poly-bir), were synthesized using established protocols in previous studies. The acid-bir was synthesized through the reduction of KMnO_4_ by HCl [1, 2]. Briefly, 65.4 mL of concentrated HCl was added to 1 L of 0.4 M boiling KMnO_4_ solution at 0.7 mL/min using a peristaltic pump. Solids formed during the HCl addition, and the suspension was stirred vigorously throughout the process. Due to HCl evaporation at the boiling temperature, KMnO_4_ was not completely consumed, as indicated by the purple color of the suspension. The delta-bir was prepared according to the method of Murray [3, 4], by reducing KMnO_4_ with Mn(NO_3_)_2_. A 750 mL of 0.15 M Mn(NO_3_)_2_ solution was pumped at 10 mL/min into a 750 mL vigorously stirred solution containing 0.1 M KMnO_4_ and 0.2 M NaOH, resulting in the generation of brown solids. The resulting suspension was aged for ~ 20 h under continuous stirring after the Mn(NO_3_)_2_ addition was complete. The poly-bir was synthesized via the reduction of KMnO_4_ by Na_2_S_2_O_3_ [5, 6]. In brief, 400 mL of a 0.188 M Na_2_S_2_O_3_ solution was pumped at 10 mL/min into 400 mL of a 0.5 M KMnO_4_ solution under vigorous stirring. The mixture was stirred vigorously for an additional 30 min after the NaS_2_O_3_ addition. All resulting suspensions were allowed to settle by gravity, and the supernatants were decanted. The slurries were then dialyzed against DI water using dialysis tubing, with the water replaced twice a day until the electrical conductivity (EC) of the slurries decreased to 14.0, 9.0, and 16.2 μS/cm for acid-bir, delta-bir, and poly-bir, respectively. The final slurries were collected and stored at 4 °C in a refrigerator prior to use. The Mn concentrations in the solids were determined to be 7.0, 6.6, and 6.6 mmol/g for acid-bir, delta-bir, and poly-bir, respectively.

**Text S2 Quantitative characterizations of birnessite precursors**

The specific surface area (SSA) of birnessite precursors were characterized using N_2_ adsorption-desorption method at liquid nitrogen temperature (~196 °C) with a gas sorption analyzer (Micromeritics ASAP 2020 instrument). Samples were degased at 105 °C for 2 h prior to the measurement. The SSA was calculated by the Brunauer-Emmett-Teller (BET) method [7].

The hydrodynamic particle sizes of three precursors were measured through dynamic light scattering using a Zetasizer Nano Series instrument (Malvern Instruments, Westborough, MA), equipped with a He−Ne laser (λ = 633 nm) and operated at a scattering angle of 90°. Samples were examined using the 1-cm disposable plastic cuvettes at 22 °C. The viscosity was set as 0.954 mPa·s and the refractive index was 1.330. The real and imaginary parts of the refractive index for birnessite were set to 2.475 and 0.01, respectively [6].

**Text S3 Quantitative spectral analysis**

Peak profile fitting was conducted for the SR-XRD patterns of the three precursors for the determination of full width at half maximum (FWHM) values for (001) and (20,11) diffraction (reflection along *c*-axis) peaks. The obtained values were employed for the calculation of coherent scattering domain size in the *ab* plane and along the *c*-axis using the Scherrer equation. The number of stacking layers of three birnessite precursors was evaluated according to the CSD size along the *c*-axis with an assumption of the basal *d*-spacing of birnessite as 7.2 Å.

Quantitative band fitting of Raman spectra was conducted for the transformation products using Jandel Scientific PeakFit 4.12 software. The raw spectra were processed with linear baseline correction and Loess smoothing using software-recommended parameters (generally below 0.5%) to prevent spectral distortion. The fitting was performed using the AutoFit Peaks I Residuals mode with Gauss Amp peak function.


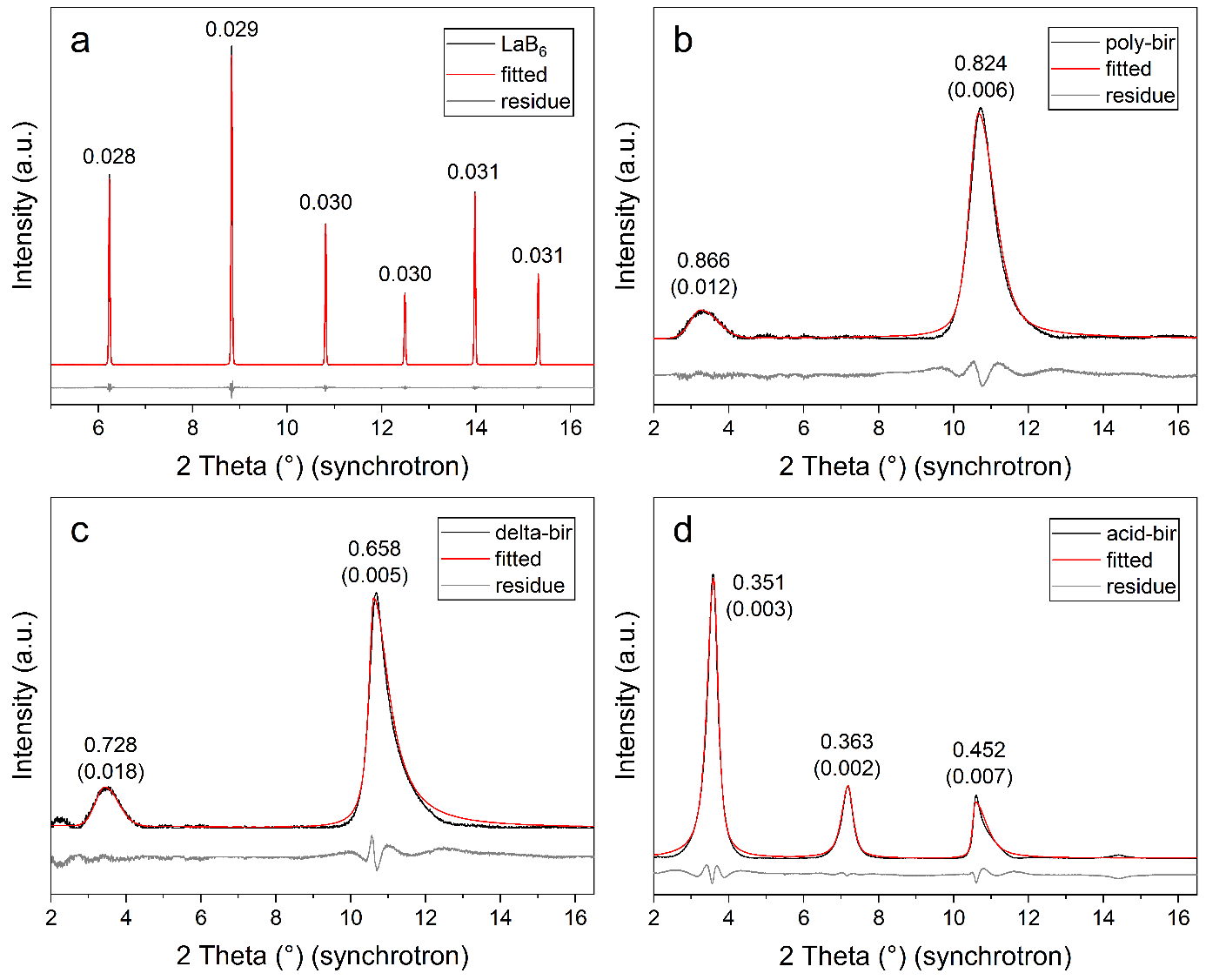


**Fig. S1** Peak profile fitting of SR-XRD patterns of LaB_6_ (a) and the synthetic birnessite precursors of varying particle sizes, i.e., poly-bir (b), delta-bir (c), and acid-bir (d), for the determination of the full width at half maximum (FWHM) and the coherent scattering domain (CSD) sizes. The FWHM values together with the variations obtained from the fittings were labeled in the panels along with the diffraction peaks. The pattern of the LaB_6_ reference sample was fitted to extract the instrumental broadening factor *β*_inst_, which was used to calibrate the physical broadening of each sample. The fitting results and calculated CSD sizes were summarized in Table 1.


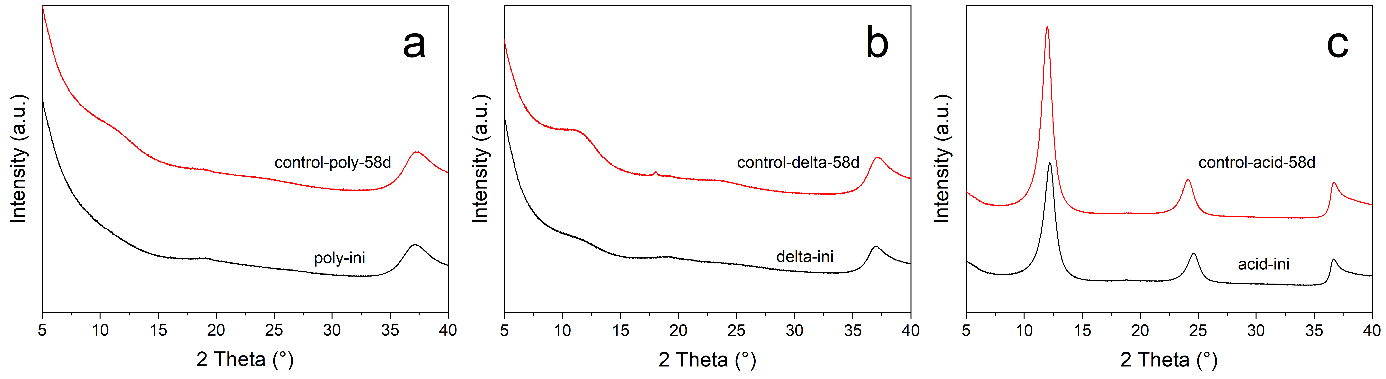


**Fig. S2** SR-XRD patterns of products derived from control experiments of birnessite incubation in the absence of aqueous Mn(II) at pH 4 for 58 days. (a) poly-bir, (b) delta-bir, and (c) acid-bir.


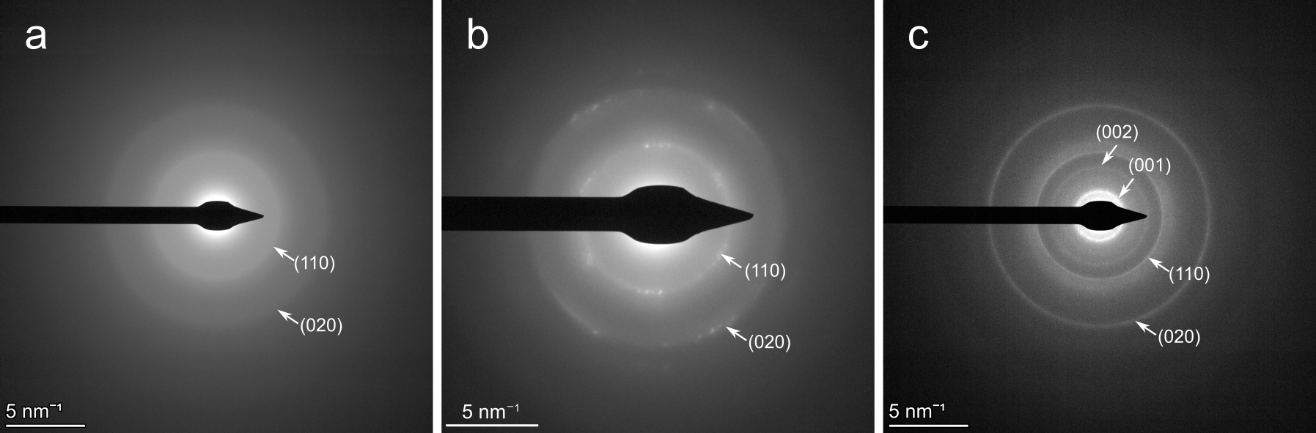


**Fig. S3** Selected area electron diffraction (SAED) patterns of the synthetic layered birnessite precursors. (a) poly-bir, (b) delta-bir, and (c) acid-bir.


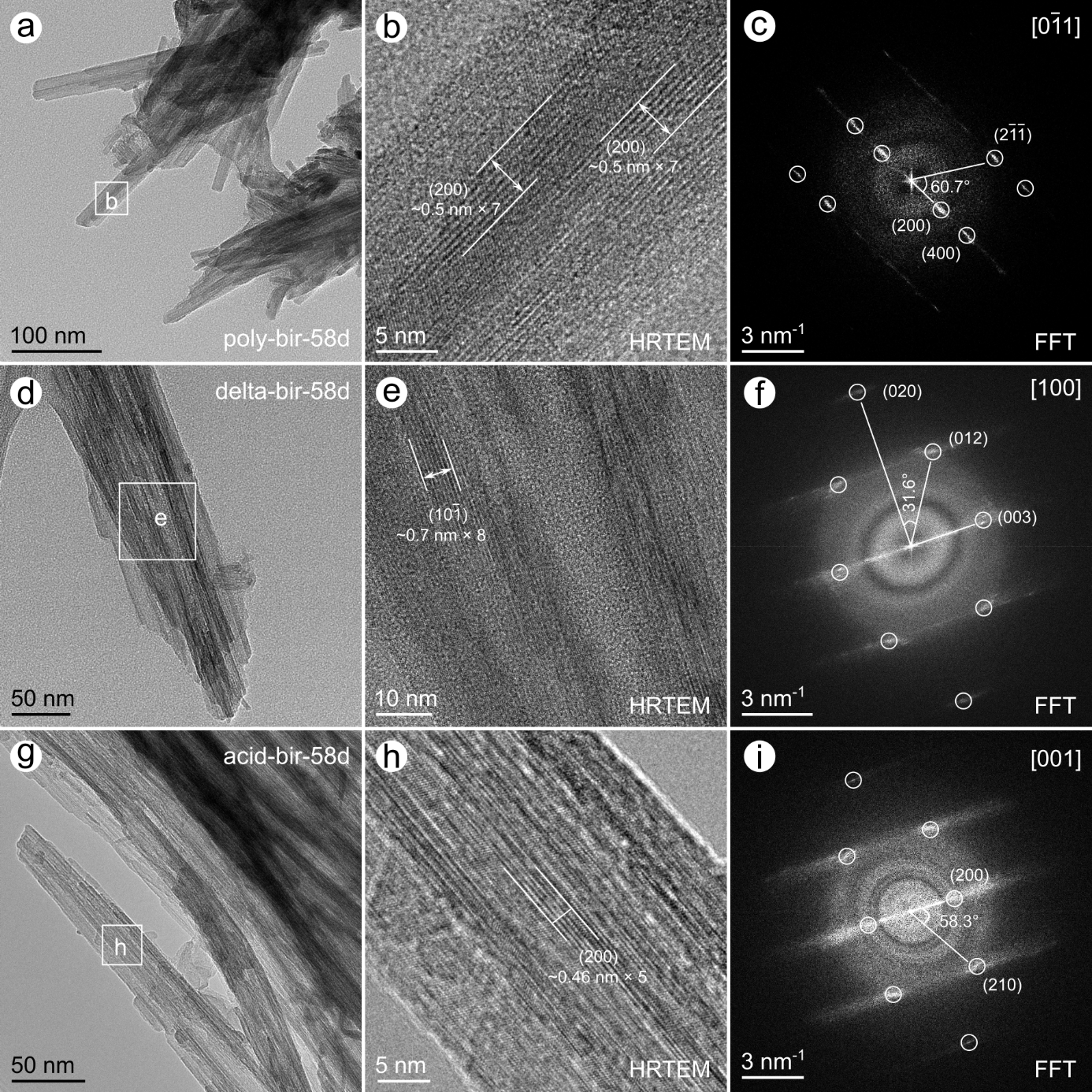


**Fig. 4** (HR)TEM images and corresponding FFT of the transformation products at 58 days. (a-c) poly-bir-58d, (d-f) delta-bir-58d, and (g-i) acid-bir-58d.


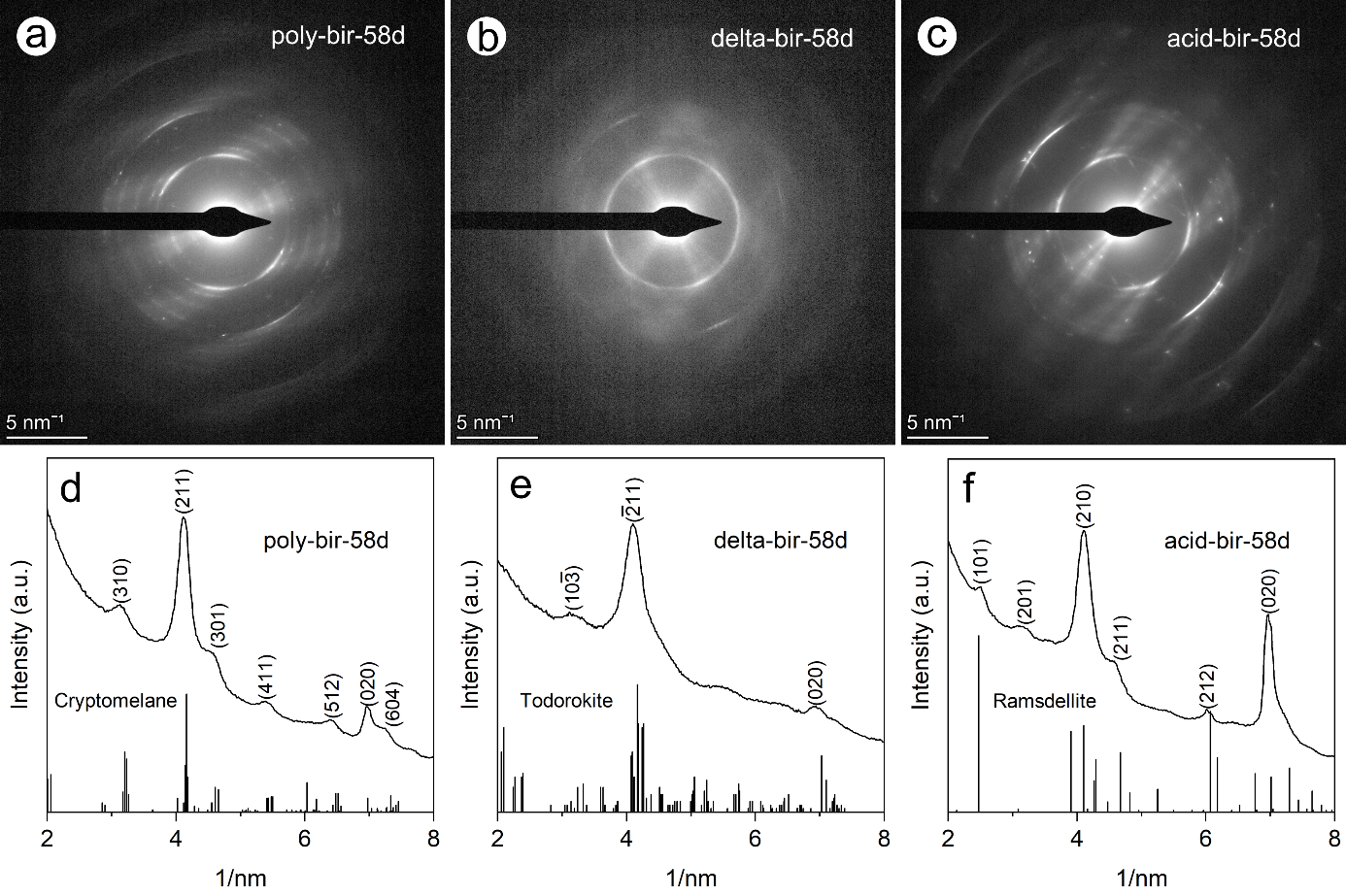


**Fig. S5** SAED patterns (a-c) and their corresponding profiles (d-f) of the transformation products derived from the incubation of birnessite precursors of varying particle sizes, with aqueous Mn(II) at Mn(II)/MnO_2_ molar ratio of 4.26 at pH 4 for 58 days.


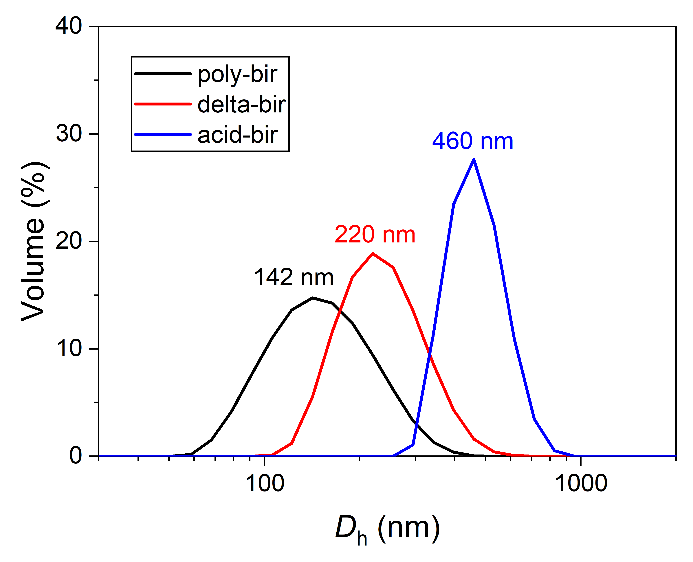


**Fig. S6** Dynamic light scattering (DLS) measured hydrodynamic particle size of the synthetic precursors of poly-bir, delta-bir, and acid-bir.


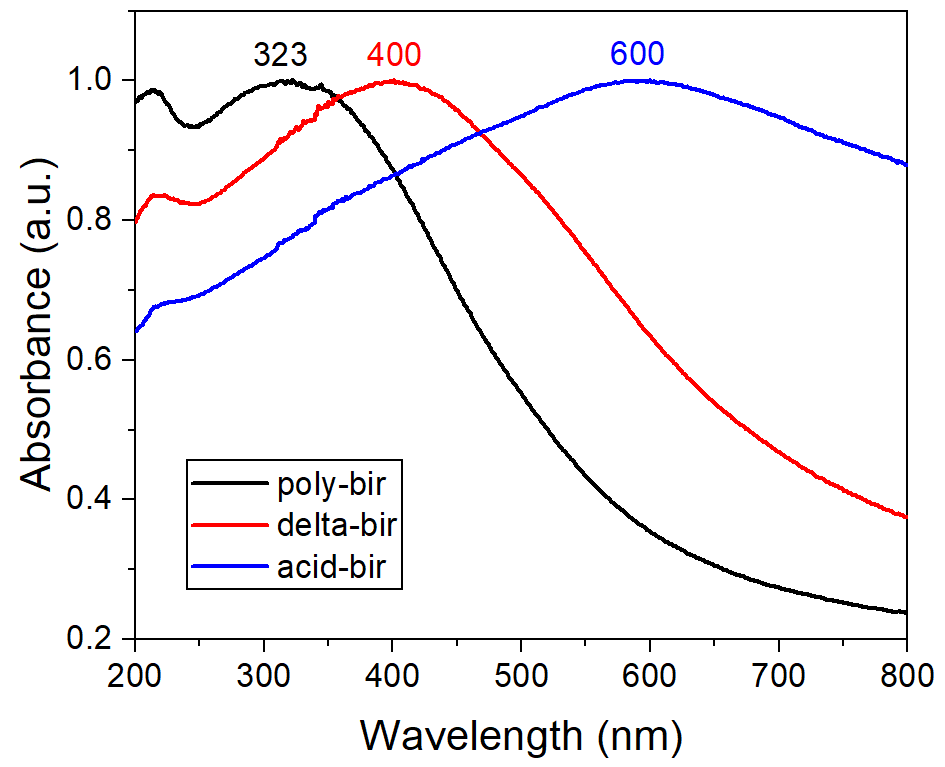


**Fig. S7** Normalized UV-vis absorption spectra for the synthetic precursors of poly-bir, delta-bir, and acid-bir in water.

**References**

1. Zhu M, Farrow CL, Post JE et al*.* Structural study of biotic and abiotic poorly-crystalline manganese oxides using atomic pair distribution function analysis. *Geochim Cosmochim Acta* 2012; **81**: 39–55.

2. Wang Q, Liao X, Xu W et al*.* Synthesis of birnessite in the presence of phosphate, silicate, or sulfate. *Inorg Chem* 2016; **55**: 10248–10258.

3. Murray JW. The interaction of metal ions at the manganese dioxide-solution interface. *Geochim Cosmochim Acta* 1975; **39**: 505–519.

4. Yang P, Wen K, Beyer KA et al*.* Mn(II)-induced phase transformation of Mn(IV) oxide in seawater. *Geochim Cosmochim Acta* 2025; **393**: 155–169.

5. Perez-Benito JF, Brillas E, Pouplana R. Identification of a soluble form of colloidal manganese(IV). *Inorg Chem* 1989; **28**: 390–392.

6. Soldatova AV, Balakrishnan G, Oyerinde OF et al*.* Biogenic and synthetic MnO_2_ nanoparticles: size and growth probed with absorption and raman spectroscopies and dynamic light scattering. *Environ Sci Technol* 2019; **53**: 4185–4197.

7. Jung H, Snyder C, Xu W et al*.* Photocatalytic oxidation of dissolved Mn^2+^ by TiO_2_ and the formation of tunnel structured manganese oxides. *ACS Earth Space Chem* 2021; **5**: 2105–2114.
